# Emergence of *Candida* (*Candidozyma*) *auris* in Minas Gerais, Brazil: Genomic Surveillance to Guide Rapid Public Health Responses

**DOI:** 10.1111/myc.70146

**Published:** 2026-02-04

**Authors:** Luiz Marcelo Ribeiro Tomé, Dhian Renato Almeida Camargo, Rafael Wesley Bastos, Sara Cândida Ferreira dos Santos, Natália Rocha Guimarães, Sílvia Helena Sousa Pietra Pedroso, Paulo Eduardo de Souza da Silva, Aristóteles Góes‐Neto, Lida Jouca de Assis Figueredo, Gabriella da Côrte Castro, Ana Maria Ribeiro Nunes Rodrigues, Flavia Ribeiro Soares Cruzeiro, Nádia Aparecida Campos Dutra, Josiane Barbosa Piedade Moura, Glauco de Carvalho Pereira, Carmem Dolores Faria, Marta Giovanetti, Felipe Campos de Melo Iani, Luiz Carlos Júnior Alcantara, Talita Émile Ribeiro Adelino

**Affiliations:** ^1^ Central Public Health Laboratory of Minas Gerais (Lacen‐MG) Ezequiel Dias Foundation—Funed Belo Horizonte Minas Gerais Brazil; ^2^ Instituto de Ciências Biomédicas (ICB) Universidade Federal do Rio de Janeiro (UFRJ) Rio de Janeiro Brazil; ^3^ Instituto René Rachou—Fiocruz Minas Belo Horizonte Minas Gerais Brazil; ^4^ Graduate Program in Bioinformatics Universidade Federal de Minas Gerais—UFMG Belo Horizonte Minas Gerais Brazil; ^5^ Centro de Biociências Universidade Federal do Rio Grande do Norte—UFRN Natal Rio Grande do Norte Brazil; ^6^ Secretaria de Estado de Saúde de Minas Gerais—SES‐MG Belo Horizonte Minas Gerais Brazil; ^7^ Department of Sciences and Technologies for Sustainable Development and One Health Universita Campus Bio‐Medico di Roma Rome Italy; ^8^ CT Vacinas—Universidade Federal de Minas Gerais Belo Horizonte Minas Gerais Brazil

**Keywords:** antifungal resistance, *Candida auris*, genomic surveillance, hospital outbreak, whole‐genome sequencing

## Abstract

**Background:**

*Candida* (*Candidozyma*) *auris* is an emerging yeast that poses a significant global health threat due to its multidrug resistance and ability to cause healthcare‐associated outbreaks. Genomic surveillance is essential for monitoring spread, transmission and antifungal resistance.

**Objectives:**

To report the first identification and genomic characterisation of *C. auris* in the state of Minas Gerais, Southeast Brazil, and to investigate the genetic origin and diversity, resistance‐associated mutations, and potential transmission dynamics during a hospital outbreak.

**Methods:**

Eight *C. auris* isolates were collected during a hospital outbreak in Belo Horizonte, Minas Gerais, Brazil, including clinical samples from patients and environmental samples from surfaces in the Intensive Care Unit (ICU). Epidemiological investigation, whole‐genome sequencing (WGS) and phylogenomic analyses were conducted to determine circulating clade, genetic diversity, outbreak origin and the presence of antifungal resistance mutations.

**Results:**

All isolates were classified as clade IV and exhibited high genomic similarity to strains previously reported in northern Colombia (Caribbean coast). One isolate carried the *ERG11* Y132F mutation, associated with fluconazole resistance, but this mutation was absent in another isolate from the same patient collected 1 day earlier, indicating mixed fungal populations. Environmental isolates clustered tightly with clinical strains, supporting surface‐mediated transmission in the ICU.

**Conclusions:**

This study describes the introduction and local spread of clade IV *C. auris* in Minas Gerais, Brazil. The findings underscore the critical role of genomic surveillance in identifying resistance mechanisms, tracing transmission pathways and guiding public health responses.

## 1 Introduction

*Candida auris* (recently renamed to *Candidozyma auris*) is an emerging multidrug‐resistant (MDR) fungal pathogen that presents a significant challenge to global public health [1, 2, 3]. First identified in Japan in 2009, *C. auris* now circulates worldwide, raising concerns due to its resistance to antifungal treatments, high transmissibility and persistence in healthcare settings [1, 4, 5].

The evolutionary origin of this pathogen remains under active investigation. However, hypotheses suggest that *C. auris* may have emerged as a human pathogen due to global warming driven by anthropogenic factors, which favoured the development of thermotolerance and facilitated its transition from an environmental organism to a clinical pathogen [6, 7]. Additionally, the increasing number of immunocompromised individuals may contribute to the emergence and establishment of new fungal diseases [8].

*Candida auris* can cause invasive candidiasis, affecting the bloodstream (candidemia), heart, central nervous system, eyes, bones and various internal organs [9, 10]. It is particularly concerning due to its high mortality rates among vulnerable patients, with case fatality ranging from 29% to 53% [10]. Infections, especially candidemia, are often associated with prolonged hospital stays compared to those caused by other *Candida* species [10].

*Candida auris* can colonise patients without causing symptoms, commonly residing on the skin, as well as in areas such as the nostrils, oropharynx, rectum and other anatomical sites. Unlike most *Candida* species, which primarily inhabit the gastrointestinal tract, *C. auris* tends to favour the skin [11, 12, 13].

Circulating strains of *C. auris* have been classified into six genomic clades, named according to their geographic region of origin: Clade I (South Asian), Clade II (East Asian), Clade III (African), Clade IV (South American), Clade V (Iranian) and Clade VI (Indomalayan) [5, 14]. This classification is based on genomic divergence, assessed through single nucleotide polymorphism (SNP) analyses, antifungal susceptibility profiles and outbreak potential [2, 5, 14, 15].

Currently, there is no consolidated global surveillance system to track suspected or confirmed *C. auris* cases and related deaths across all countries. Due to limited data and the lack of comprehensive epidemiological studies, global annual incidence rates remain undetermined. Nonetheless, a growing number of cases has been reported worldwide, underscoring the expanding spread of this pathogen across diverse regions [5, 9].

The global rise in *C. auris* cases, together with its antifungal resistance and high outbreak potential, led the Pan American Health Organization (PAHO) and the World Health Organization (WHO) to include this fungus in the WHO Fungal Priority Pathogens List (2022), created to guide research, development and public health strategies. Classified in the Critical group, *C. auris* is recognised as a major global health threat [10, 16].

In Brazil, *C. auris* was first identified in December 2020 in Salvador, Bahia, in the Northeast region. The isolate was classified as Clade I and was susceptible to key antifungal agents, including amphotericin B, fluconazole, voriconazole and anidulafungin [17, 18, 19]. By late 2021, isolates from Clade IV were detected in the state of Pernambuco, which is also in the northeast region of Brazil, demonstrating susceptibility to amphotericin B, azoles and echinocandins [20, 21]. Since then, Pernambuco has experienced multiple outbreaks [20, 21]. In 2023, *C. auris* was additionally reported for the first time in the states of Rio de Janeiro and São Paulo, both located in the Southeast region of Brazil [22]. Despite these current knowledge, significant gaps remain in our understanding of the genomic diversity, transmission patterns and epidemiological dynamics of *C. auris* within Brazil.

In September 2024, *C. auris* was detected for the first time in a hospital in Belo Horizonte, Minas Gerais, Brazil. Preliminary evidence suggested a possible introduction from Colombia. In response to the emergence of this multidrug‐resistant pathogen, fungal genomic surveillance was promptly implemented at the Central Public Health Laboratory of Minas Gerais (Lacen‐MG), based at the Ezequiel Dias Foundation (Funed).

To investigate the origin and transmission dynamics of this event, we conducted genomic and epidemiological investigations of the *C. auris* isolates collected during this outbreak. Here, we report their detection and genomic characterisation through an integrated approach that combines epidemiological data, whole‐genome sequencing, phylogenetic analysis and the identification of resistance‐associated mutations. This strategy aimed to enhance our understanding of the emergence and regional spread of *C. auris* in Southeast Brazil.

## 2 Material and Methods

### 2.1 Sample Collection and Pathogen Identification

Clinical samples, including urine and swabs from the axillary and inguinal regions, were collected from patients admitted to the ICU with suspected *C. auris* infection or colonisation at a hospital in Belo Horizonte, Minas Gerais, Brazil (Table 1). Additionally, environmental swabs were collected from ICU equipment (beds and cables) to assess potential environmental dissemination. Samples were transported to Lacen‐MG in tubes containing Sabouraud Dextrose Broth, processed and plated onto *Candida* Plus chromogenic agar (Kasvi), followed by incubation for 72 h at 37°C. Colonies suspected to be *C. auris* were identified by Matrix‐Assisted Laser Desorption Ionisation Time‐of‐Flight (MALDI‐TOF) mass spectrometry (VITEK MS—BIOMÉRIEUX). To confirm the identification, DNA from *C. auris* colonies was extracted and subjected to quantitative PCR (qPCR), following the Centers for Disease Control and Prevention (CDC) protocol [23].

**TABLE 1:** Demographic data of patients and details of *Candida auris* positive samples sequenced in this study.

Patient	Sample ID	State	Municipality	Sample source	Biological material	Patiente age (years)	Sex	Collection date
1	FUNED538^a^	Minas Gerais	Belo Horizonte	Clinical Sample	Urine	58	Male	2024‐09‐17
2	FUNED558^a^	Minas Gerais	Belo Horizonte	Clinical Sample	Axillary/inguinal swab	44	Male	2024‐09‐27
3^b^	FUNED569^a^	Minas Gerais	Belo Horizonte	Clinical Sample	Axillary/inguinal swab	38	Male	2024‐10‐03
4^b^	FUNED575^a^	Minas Gerais	Belo Horizonte	Clinical Sample	Axillary/inguinal swab	58	Male	2024‐10‐03
3^b^	FUNED593^a^	Minas Gerais	Belo Horizonte	Clinical Sample	Axillary/inguinal swab	38	Male	2024‐10‐04
4^b^	FUNED596^a^	Minas Gerais	Belo Horizonte	Clinical Sample	Axillary/inguinal swab	58	Male	2024‐10‐04
—	FUNED726^a^	Minas Gerais	Belo Horizonte	Environmental	Hospital bed (ICU‐21)	—	—	2024‐10‐11
—	FUNED727^a^	Minas Gerais	Belo Horizonte	Environmental	Cables (ICU‐21)	—	—	2024‐10‐11

^**a**^ All samples in this table tested positive for *C. auris* using both MALDI‐TOF and qPCR assays.

^**b**^ Patients had samples collected twice on different days.

### 2.2 Patient Data

Patients with *C. auris* colonisation confirmed through MALDI‐TOF and qPCR had demographic data, including municipality and state of request for examination and hospitalisation, and additional information, such as sex, age and travel history assessed. These data were obtained from the epidemiological forms sent to Lacen‐MG and through the GAL system (Laboratory Environment Management), managed by the Ministry of Health of Brazil. Clinical data, including hospitalisation details, sample collection date, sample types and antifungal therapy, were retrieved from clinical records and the Secretaria de Estado de Saúde de Minas Gerais (SES‐MG). This study was approved by the research ethics committee of the Ezequiel Dias Foundation (CAAE: 85347524.5.0000.9507).

### 2.3 Whole‐Genome Sequencing

For fungal genomic surveillance at Lacen‐MG, DNA from eight *C. auris* isolates was subjected to WGS using the MiSeq (Illumina) and Ion Torrent PGM (Thermo Fisher Scientific) platforms. For Illumina sequencing, genomic DNA from seven *C. auris* samples was used for library preparation with the Illumina DNA Prep Kit (Illumina), following the manufacturer's protocol. The final library pool was loaded onto a MiSeq Reagent Kit v3 (600 cycles, 2 × 300 bp read length) and sequenced on a MiSeq system (Illumina) at a final concentration of 8 pM.

For Ion Torrent PGM sequencing, genomic DNA from one *C. auris* sample (FUNED538) was fragmented using the Ion Shear Plus Reagents Kit (Thermo Fisher Scientific). Then, fragmented DNA was used for library preparation with the NEBNext Fast DNA Library Prep Kit (NEB) and Ion Xpress Barcode Adapters 1–16 Kit (Thermo Fisher Scientific), according to the manufacturer's recommendations. Emulsion PCR was performed to amplify the library using the Ion PGM Hi‐Q View OT2 Kit (Thermo Fisher Scientific) on the Ion OneTouch 2 system (Thermo Fisher Scientific). Ion sphere particles were enriched using the Ion OneTouch ES system (Thermo Fisher Scientific) and subsequently sequenced on an Ion 318 chip with the Ion PGM Hi‐Q View Sequencing Kit on the Ion Torrent PGM platform (Thermo Fisher Scientific).

### 2.4 Genome Assembly and Quality Assessment

Raw sequencing data (FASTQ files) from the eight *C. auris* isolates obtained in this study were initially assessed for quality using FastQC (v0.12.1—github.com/s‐andrews/FastQC) and subsequently trimmed with Trimmomatic (v0.39—github.com/usadellab/Trimmomatic) to remove low‐quality bases and adapter sequences. Genome assembly was performed with the software SPAdes (v4.0.0) [24]. Misassemblies were corrected, and contigs were scaffolded using RagTag (v2.1.0—github.com/malonge/RagTag) with a reference *C. auris* genome (GCA_041381755.1) obtained from GenBank (NCBI). Assemblies were further polished with Pilon (v1.24). The quality and completeness of the assembled genomes were evaluated using QUAST (v5.2.0—github.com/ablab/quast) and BUSCO (v5.4.7—github.com/metashot/busco), respectively.

To conduct a comprehensive genomic surveillance study and include Clade IV genomes of *C. auris* isolates from Brazil, we retrieved raw sequencing data (FASTQ files) for six isolates associated with outbreaks in Recife, Pernambuco, during 2022 and 2023 [21]. These data were retrieved from BioProject ID PRJNA1055119 in the NCBI SRA database. The sequences were subjected to whole‐genome assembly using the same tools and analytical pipeline applied to the eight isolates obtained in this study from Minas Gerais.

### 2.5 Phylogenomic and SNP‐Based Analyses

To construct a phylogenetic tree, the BUSCO phylogenomics pipeline (github.com/jamiemcg/BUSCO_phylogenomics) was employed to align 978 single‐copy orthologous proteins from 150 publicly available *C. auris* genomes (Clades I–VI) retrieved from GenBank (NCBI) (Table S1), along with eight genomes sequenced in this study. IQ‐TREE (v2.3.6) was subsequently used to determine the best‐fitting evolutionary model and to infer the maximum likelihood phylogeny.

The software Parsnp (v1.2; github.com/marbl/parsnp) was used to perform core genome alignment, detect single nucleotide polymorphisms (SNPs), and generate a second phylogenetic tree based on all 158 *C. auris* genomes. Variants identified by Parsnp were exported as a VCF file using Gingr (v1.3) and analysed using R software to perform Principal Component Analysis (PCA) and generate a heatmap of SNP differences among Clade IV isolates. Only variants that passed the internal quality filters (FILTER = PASS) were retained to ensure reliable SNP calls.

### 2.6 Analysis of Resistance‐Associated Genes

To investigate antifungal resistance, the eight genomes generated in this study were analysed for mutations in key genes associated with resistance: *ERG11* (encoding lanosterol 14 α‐demethylase), *ERG6* (encoding sterol 24‐C‐methyltransferase), *FKS1* (encoding 1,3‐beta‐glucan synthase), *TAC1B* (encoding a zinc‐cluster transcription factor) and *ERG2* (encoding C‐8 sterol isomerase) [14, 21, 25]. Know missense mutations linked to antifungal resistance were identified using Blastn (NCBI) to locate target regions, MAFFT (v7.490) for sequence alignment and AliView for alignment inspection. Detected mutations were validated by mapping sequencing reads to the corresponding gene regions to assess the percentage of mapped reads, coverage and depth.

## 3 Results

### 3.1 Outbreak Detection and Early Investigation in Minas Gerais

**FIGURE 1:**
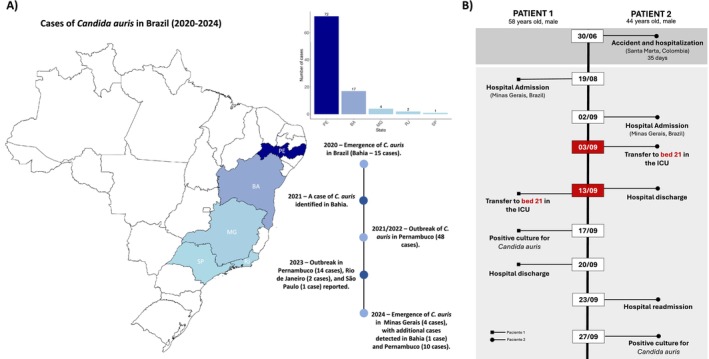
Overview of the emergence of *Candida auris* in Belo Horizonte, Minas Gerais, Brazil. (A) Map of Brazil indicating reported *C. auris* cases and outbreaks from 2020 to 2024 across different states. (B) Timeline detailing the first *C. auris* cases identified in a hospital in Minas Gerais. Patient 1 was admitted on August 19, 2024, for an aortic dissection and transferred to ICU bed 21 on September 13. The patient was discharged from the ICU on September 16, and on September 17, a urine culture tested positive for *C. auris* . Patient 2, who had been hospitalised for 35 days following an accident in Santa Marta, Colombia, was admitted to the same hospital in Brazil on September 2, underwent surgery on September 3, and was also placed in ICU bed 21.

The emergence of *C. auris* in Brazil was first documented in 2020 during the COVID‐19 pandemic, and the pathogen has since been detected in five Brazilian states (Figure 1a). Its occurrence in geographically distant regions suggests ongoing healthcare‐associated transmission and possibly multiple independent introductions. Within this national context, by late 2024 an outbreak was detected in Belo Horizonte, Minas Gerais, representing the first documented occurrence of *C. auris* in the state (Figure 1a).

The initial case of *C. auris* in Minas Gerais was identified in a patient (Patient 1) admitted to the ICU of a public hospital in Belo Horizonte on August 19, 2024 (Table 1, Figure 1b). A clinical specimen collected on September 17, 2024, tested positive for *C. auris* by matrix‐assisted laser desorption/ionisation time‐of‐flight mass spectrometry (MALDI‐TOF MS) and qPCR. Routine active surveillance subsequently identified a second colonised patient on September 27, 2024 (Figure 1b).

In response, the hospital initiated an epidemiological investigation in collaboration with the Minas Gerais State Health Department (Secretaria de Estado de Saúde de Minas Gerais—SES‐MG) to elucidate the potential source and transmission dynamics. All colonised patients were male, aged 38–58, and hospitalised in the same ICU (Table 1).

Epidemiological mapping implicated ICU bed 21 as the likely focal point of *C. auris* spread (Figure 1b). Notably, this bed had been previously occupied by a patient (Patient 2) transferred from Santa Marta, Colombia, following hospitalisation in that country. This patient was admitted to the same hospital and assigned to bed 21 shortly before the admission of the first confirmed case (Patient 1), indicating a potential index patient and source of introduction.

On October 3 and 4, 2024, two additional ICU patients were confirmed positive for *C. auris* colonisation. Environmental sampling conducted on October 11, 2024, yielded positive results for *C. auris* on surfaces associated with bed 21, including monitoring cables, confirming environmental contamination and supporting the hypothesis of nosocomial transmission originating from this site. Notably, all cases reported in this study up to that point represented colonisation, with no invasive infections observed.

### 3.2 Whole‐Genome Characterisation of the Minas Gerais Isolates

**FIGURE 2:**
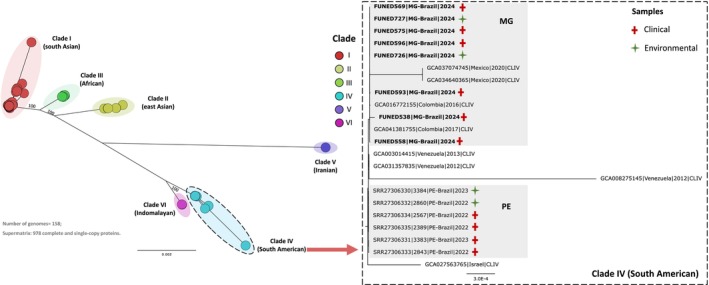
Maximum likelihood phylogenetic tree based on 978 concatenated proteins from 158 *Candida auris* genomes, showing that isolates from Belo Horizonte cluster within clade IV. The tree was constructed using IQ‐TREE under the JTTDCMut+F + R3 model with 1000 bootstrap replicates. Enlarged clade and highlighted subclades indicate that Minas Gerais (MG) isolates cluster within clade IV and are clearly distinct from Pernambuco outbreak (PE) isolates.

**FIGURE 3:**
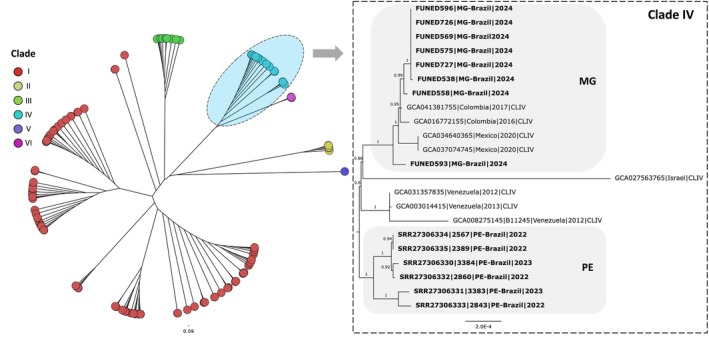
Phylogenetic tree of 158 *Candida auris* genomes based on core genome alignment and SNP detection using Parsnp. The enlarged clade and highlighted subclades show that Minas Gerais (MG) isolates cluster within clade IV (South American) and are distinct from Pernambuco outbreak (PE) isolates. Support values are indicated for the major clades.

**FIGURE 4:**
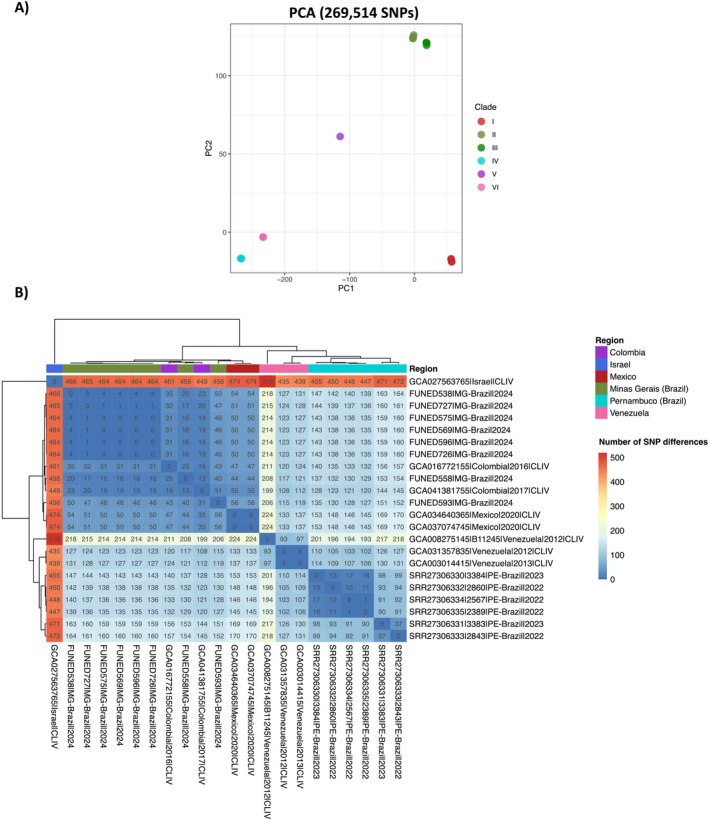
Genomic relationships among *Candida auris* isolates based on SNP data. (A) Principal Component Analysis (PCA) based on 269,514 SNPs from 158 genomes, showing genetic separation among clades. (B) Heatmap showing SNP differences among Clade IV isolates.

Using WGS, we obtained complete genomes of clinical and environmental *C. auris* isolates from Minas Gerais (Table 2). The assembled genomes ranged from 12,228,794 to 12,342,789 bp, encompassing seven nuclear chromosomes and a complete mitochondrial genome, with completeness between 98.3% and 99% based on single‐copy ortholog analysis using BUSCO. Phylogenomic analysis based on 978 concatenated proteins placed all isolates from Minas Gerais within Clade IV, clustering closely with genomes from Colombia and Mexico (Figure 2). SNP‐based analysis further supported this placement, with all isolates grouped within their respective clades in both the SNP phylogenetic tree and PCA plot (Figures 3 and 4a).

**TABLE 2:** Genomic metrics of *Candida auris* genomes from isolates collected in Belo Horizonte, Minas Gerais, Brazil.

Sample ID	*Reads* sequenced	Beses sequenced	Sequencing depth	Genome length (bp)	Number of scaffolds (chromosomes)	N50	L50	GC (%)	Complete BUSCOs^a^(%)
FUNED538	5,038,765	1,020,284,526	82	12,266,768	7	2,306,824	2	45.18	98.3
FUNED558	7,076,859	1,300,586,893	106	12,228,794	7	2,320,025	2	45.12	98.5
FUNED569	3,404,286	652,662,068	53	12,342,789	7	2,317,092	2	45.13	99.0
FUNED575	5,493,388	1,025,455,581	83	12,332,892	7	2,316,038	2	45.12	99.0
FUNED593	4,296,743	815,830,067	66	12,279,414	7	2,307,669	2	45.12	99.0
FUNED596	4,563,919	873,120,734	70	12,327,528	7	2,315,396	2	45.12	99.0
FUNED726	10,059,594	1,840,102,376	149	12,326,563	7	2,316,074	2	45.11	99.0
FUNED727	8,052,864	1,520,151,176	123	12,336,396	7	2,315,903	2	45.12	99.0

^a^ BUSCO analysis based on the presence of complete single‐copy orthologous genes.

The SNP tree, generated from core‐genome alignment using Parsnp (Figure 3), confirmed the close relationship between Minas Gerais and Colombian isolates, suggesting a potential introduction into Belo Horizonte from Colombia. Notably, the genome from Patient 2 (FUNED558), who had a history of hospitalisation in Santa Marta, Colombia, appeared as an early branch within this cluster, indicating it may represent the index case. Retrospective data show that *C. auris* has been circulating in Santa Marta since at least 2012 [26]. Additionally, all Minas Gerais isolates carry amino acid substitutions in the *ERG11* gene (K177R, N335S, E343D), consistent with Colombian strains [27]. It is noteworthy that the genome of isolate FUNED593 clustered separately from the other isolates from Minas Gerais in the SNP‐based phylogenetic tree, possibly reflecting the coexistence of distinct fungal subpopulations.

The genomes from Pernambuco formed a distinct monophyletic group (PE clade) in both protein‐ and SNP‐based phylogenetic trees (Figures 2 and 3), clearly separated from the Minas Gerais isolates (MG clade), with a bootstrap value of 1. These findings strongly support the hypothesis of an independent introduction event in Minas Gerais, unrelated to the Pernambuco outbreak.

Further exploration of the Clade IV cluster, based on core genome alignment and SNP difference analysis, revealed a high degree of genomic relatedness among the Minas Gerais isolates, encompassing both clinical and environmental samples (Figure 4b). Among them, isolate FUNED593 emerged as the most divergent, displaying a distinct SNP profile compared with isolate FUNED569, which had been collected from the same patient only 1 day earlier. Importantly, this analysis not only highlights the tight genomic relationship among Minas Gerais isolates but also underscores their clear genetic separation from those in Pernambuco, pointing to distinct evolutionary trajectories underlying the two *C. auris* outbreaks.

### 3.3 Genomic Analysis of Antifungal Resistance‐Associated Genes

**FIGURE 5:**
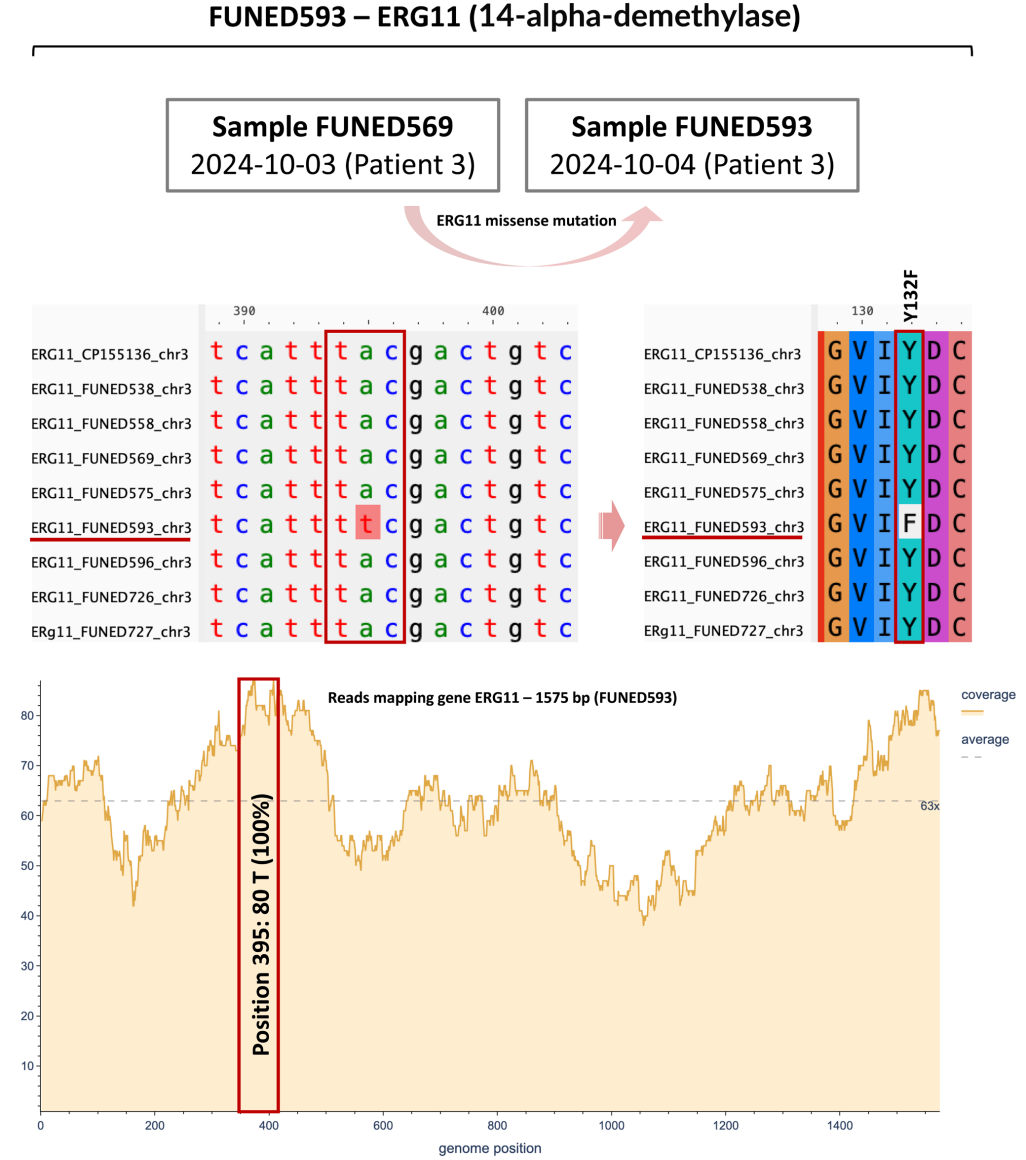
Analysis of the ERG11 gene in isolate FUNED593 showing an A‐to‐T nucleotide substitution at position 395, resulting in a Y132F amino acid change (tyrosine to phenylalanine), associated with fluconazole resistance. This mutation was absent in the previous‐day sample (FUNED569) from the same patient. Read mapping of ERG11 showed 80× coverage at the mutation site in FUNED593, confirming its presence. Sequences were aligned against chromosome 3 (CP155136.1) of genome GCA_041381755.1 (Colombia, 2017).

Genomic analysis of all *C. auris* isolates in this study revealed the presence of a missense mutation in the *ERG11* gene exclusively in the FUNED593 genome, obtained from Patient 3 (Figure 5). The identified mutation leads to a tyrosine‐to‐phenylalanine substitution at position 132 (Y132F) in *Erg11p*, an alteration well‐established as a mechanism of fluconazole resistance in *C. auris* and other *Candida* species (Figure 5) [28, 29]. Notably, this mutation was absent in a previous sample collected from the same patient just 1 day earlier.

## 4 Discussion

The first case of *C. auris* in Brazil was reported in December 2020, during the COVID‐19 pandemic, in Salvador, Bahia, with 15 cases and two associated deaths recorded that year (Figure 1a) [17, 22]. By the end of 2021, only one additional case was reported nationwide. However, by late 2021 and throughout 2022, an outbreak involving 48 cases emerged in Pernambuco, affecting two hospitals and accounting for a significant proportion of the country's cases [21, 22]. In 2023, Pernambuco reported 14 new cases across six hospitals, highlighting both the ongoing transmission and the pathogen's potential to cause multicentric outbreaks [22].

Also in 2023, *C. auris* was identified for the first time in Brazil's Southeastern region, with two cases in Rio de Janeiro and one in São Paulo, the latter involving a newborn (Figure 1a) [22]. In 2024, new cases were detected in Pernambuco (10 cases) and Bahia (1 case) [22]. By the end of that year, an outbreak was identified in Belo Horizonte, Minas Gerais (Figure 1a), marking the first known occurrence of the pathogen in the state.

In this study, we report for the first time the identification of *C. auris* in the city of Belo Horizonte, in the state of Minas Gerais, Brazil, and implemented genomic surveillance of pathogenic fungi to investigate and characterise the introduction of this etiological agent into the state. WGS of eight *C. auris* isolates from Minas Gerais revealed that all belonged to clade IV (South American), showing the greatest genomic similarity to isolates previously described in Colombia (Figures 2, 3, 4).

The integration of genomic, epidemiological, and clinical data supported the hypothesis that the emergence of the pathogen in Minas Gerais may be related to a patient with a history of prior hospitalisation in Santa Marta, a city located on the northern coast of Colombia (Figures 1, 2, 3). Although laboratory surveillance of *C. auris* in Colombia was officially implemented in 2016, retrospective analyses identified the presence of the fungus as early as 2012 in samples from Santa Marta [26].

This hypothesis is further supported by phylogenetic analyses, which showed that the *C. auris* genomes from Minas Gerais form a distinct cluster from those isolated in the state of Pernambuco (Figures 2, 3, 4). The Colombian genomes most closely related to the isolates from Minas Gerais were obtained from Cartagena, located on the northern coast of Colombia, and Valledupar, a city in northern Colombia. This geographic proximity reinforces the possible link between the introduction of the pathogen in Minas Gerais and the patient's prior hospitalisation in Santa Marta, also situated on Colombia's northern coast.

In Brazil, genomic surveillance of *C. auris* remains limited, as reflected by the small number of complete genomes available in public databases. Although the pathogen was first reported in the country in 2020, and 96 cases had been documented by December 2024, only one genome, derived from the initial outbreak and belonging to clade I (South Asian), had been deposited in the NCBI [17, 29]. In early 2025, raw WGS data from six isolates from Pernambuco became publicly available, allowing us to assemble these genomes using the same pipeline applied to the Minas Gerais isolates, thereby enabling consistent comparisons and robust phylogenetic inference [21]. However, outbreaks reported in other states, such as Rio de Janeiro and São Paulo, still lack available genomic data, which hinders broader and more effective genomic and epidemiological surveillance.

In this study, analysis of key genes relates to antifungal resistance (*ERG11*, *TAC1B*, *ERG2*, *ERG6* and *FKS1*) across the eight genomes revealed a missense mutation in the *ERG11* gene of an isolate from Patient 3 (FUNED593). This mutation consists of an adenine‐to‐thymine substitution at position 395, resulting in a tyrosine‐to‐phenylalanine substitution at amino acid position 132 (Y132F) (Figure 5). This mutation is well documented in the literature and associated with azole resistance [5, 25, 28, 30]. Interestingly, a sample collected the previous day (October 3, 2024) from the same patient (FUNED569) did not exhibit this mutation, suggesting either a pre‐existing mixed population or the recent emergence of the resistant variant.

To investigate this finding, we performed additional bioinformatic analyses, including read mapping of sample FUNED593 to the *ERG11* gene sequence (Figure 5), to rule out sequencing or assembly errors. The results showed a sequencing coverage depth of 80× at the mutation site, with all mapped reads displaying the thymine (T) base, confirming the nucleotide substitution and ruling out technical error. This is the first report of the Y132F mutation in a Brazilian *C. auris* isolate. Notably, the patient was not receiving antifungal treatment at the time of sample collection, which at least directly excludes pharmacological selective pressure as a determining factor in the emergence of the mutation.

This finding raises the possibility of other, as‐yet‐unidentified selective pressures that may have contributed to the appearance of this mutation. It is important to note that the patient from whom the mutated isolate was obtained died, although the death was not attributed to *C. auris* infection. A limitation of this study, however, is the absence of antifungal susceptibility testing (AFST), which limits direct confirmation of the phenotypic impact of the Y132F mutation. Nevertheless, the genomic evidence presented here provides a strong indication of its potential role in fluconazole resistance.

SNP‐based phylogenetic tree and the pairwise SNP distance heatmap revealed that the genome of sample FUNED593 differs from both FUNED569 and the other isolates from Minas Gerais (Figures 3 and 4). FUNED593 also diverged from Colombian genomes (GCA041381755|2017 and GCA016772155|2016), all belonging to clade IV, as illustrated by the pairwise SNP distance heatmap (Figure 4). Notably, when compared to FUNED569, obtained from the same patient 1 day earlier, 46 SNPs were detected (Figure 4), suggesting a pre‐existing mixed fungal population.

As part of this study, environmental samples were also collected from the bed and monitor cables in the ICU where the outbreak occurred in Belo Horizonte. Genomic analysis of these isolates showed that they clustered with clinical isolates from the same outbreak, exhibiting low genetic divergence in phylogenetic trees based on single‐copy orthologous proteins and SNPs, as well as in the pairwise SNP distance heatmap (Figures 2, 3, 4). These results indicate likely transmission via contaminated ICU surfaces. The detection of *C. auris* on environmental surfaces such as beds and monitoring equipment highlights the critical role of environmental contamination in the persistence and dissemination of the pathogen in healthcare settings.

## 5 Conclusions

This study reports the first detection of *C. auris* in Belo Horizonte, Minas Gerais, likely introduced from Colombia, with genomic evidence supporting hospital‐based transmission. Phylogenomic analyses identified key genomic characteristics and emerging mutations linked to antifungal resistance. These findings reinforce the crucial role of genomic surveillance in tracking pathogen dissemination and resistance mechanisms, thereby informing timely and effective public health responses. They also demonstrate that *C. auris* colonisation alone can drive the rapid emergence of resistance mutations, underscoring the need for rigorous hospital monitoring and containment measures. Moreover, the open sharing of genomic data from local outbreaks is essential to strengthen ongoing surveillance efforts and to support future research aimed at the development of novel diagnostics, antifungal agents and therapeutic targets.

## Author Contributions

**Luiz Marcelo Ribeiro Tomé:** conceptualisation, data curation, formal analysis, investigation, methodology, validation, visualisation, writing – original draft preparation and writing – review and editing. **Dhian Renato Almeida Camargo:** investigation and writing – review and editing. **Rafael Wesley Bastos:** formal analysis and writing – review and editing. **Sara Cândida Ferreira dos Santos:** data curation and writing – review and editing. **Natália Rocha Guimarães:** data curation, visualisation and writing – review and editing. **Sílvia Helena Sousa Pietra Pedroso:** data curation, visualisation and writing – review and editing. **Paulo Eduardo de Souza da Silva:** investigation and writing – review and editing. **Aristóteles Góes‐Neto:** resources and writing – review and editing. **Lida Jouca de Assis Figueredo:** investigation and writing – review and editing. **Gabriella da Côrte Castro:** investigation and writing – review and editing. **Ana Maria Ribeiro Nunes Rodrigues:** investigation and writing – review and editing. **Flavia Ribeiro Soares Cruzeiro:** investigation and writing – review and editing. **Nádia Aparecida Campos Dutra:** investigation and writing – review and editing. **Josiane Barbosa Piedade Moura:** investigation and writing – review and editing. **Glauco de Carvalho Pereira:** investigation and writing – review and editing. **Carmem Dolores Faria:** investigation and writing – review and editing. **Marta Giovanetti:** investigation and writing – review and editing. **Felipe Campos de Melo Iani:** investigation and writing – review and editing. **Luiz Carlos Júnior Alcantara:** resources and writing – review and editing. **Talita Émile Ribeiro Adelino:** formal analysis, investigation, methodology, validation, writing – review and editing.

## Conflicts of Interest

The authors declare no conflicts of interest.

## Supporting information


**Table S1:** Accession numbers for C. auris genomes obtained from NCBI (Assembly/Genomes) and raw whole‐genome sequencing data obtained from the Sequence Read Archive (SRA), used for phylogenetic analyses, SNP analysis, clade assignment, and identification of antifungal resistance‐associated genes.

## Data Availability

The Whole Genome Shotgun (WGS) projects generated in this study have been deposited at DDBJ/ENA/GenBank under BioProject accession number PRJNA1194488 (Genomic and Epidemiological Surveillance of Candida auris in Minas Gerais, Brazil). The genome assemblies are available under the following accession numbers: JBORIE000000000 (SAMN47863631/FUNED575), JBORID000000000 (SAMN47863578/FUNED569), JBORIC000000000 (SAMN47862471/FUNED558), JBNLHW000000000 (SAMN47871007/FUNED727), JBNLHV000000000 (SAMN47869215/FUNED726), JBNLHU000000000 (SAMN47869145/FUNED596), JBNLHT000000000 (SAMN47869045/FUNED593) and JBMWAV000000000 (SAMN47784825/FUNED538).
